# *In situ* viscoelastic properties and chain conformations of heavily hydrated carboxymethyl dextran layers: a comparative study using OWLS and QCM-I chips coated with waveguide material

**DOI:** 10.1038/s41598-018-30201-6

**Published:** 2018-08-07

**Authors:** Andras Saftics, György Aurél Prósz, Barbara Türk, Beatrix Peter, Sándor Kurunczi, Robert Horvath

**Affiliations:** 1 Nanobiosensorics Laboratory, Centre for Energy Research, Hungarian Academy of Sciences, Konkoly Thege Miklós út 29-33, Budapest, 1121 Hungary; 2 Faculty of Chemical Technology and Biotechnology, Budapest University of Technology and Economics, Műegyetem rkp. 3, Budapest, 1111 Hungary

## Abstract

Hydration, viscoelastic properties and dominant structure of thin polymer layers on the surface of waveguide material were evaluated using optical waveguide lightmode spectroscopy (OWLS) and quartz crystal microbalance (QCM) methods. The fundamentally different principles of the two applied label-free biosensors enable to examine analyte layers from complementary aspects, e.g. to determine the amount of bound water in hydrated layers. In this study, a new QCM instrument with impedance measurement (QCM-I) is introduced. Its specially designed sensor chips, covered by thin film of waveguide material, supply identical surface as used in OWLS sensors, thus enabling to perform parallel measurements on the same type of surface. Viscoelastic analysis of the measured data was performed by our evaluation code developed in MATLAB environment, using the Voinova’s Voigt-based model. *In situ* deposition experiments on the ultrathin films of poly(L-lysine)-*graft*-poly(ethylene glycol) (PLL-*g*-PEG) were conducted for instrumental and code validation. Additionally, a novel OWLS-QCM data evaluation methodology has been developed based on the concept of combining hydration and viscoelastic data with optical anisotropy results from OWLS measurements. This methodology provided insight into the time-dependent chain conformation of heavily hydrated nano-scaled layers, resulting in unprecedented structural, hydration and viscoelastic information on covalently grafted ultrathin carboxymethyl dextran (CMD) films. The measured mass values as well as hydration and viscoelastic properties were compared with the characteristics of PLL-*g*-PEG layers.

## Introduction

Label-free biosensing offers a number of opportunities for exploring phenomena which have been hidden from the field of view of conventional labeling techniques. The main attractive consequence of the label-free principle is the elimination of disturbing effect and cost of label molecules as well as the ability to measure the kinetic behavior of interactions even at the sub-molecular level by allowing real-time monitoring (while the conventional labeling methods provide only end-point data of biomolecular and cellular interactions)^1^. Several label-free transduction principles have been reported and proven to be suitable for pharmaceutical industry or healthcare, i.e. for point-of-care testing, and their applications in basic researches are on the rise^2,3^. Regarding the applied transduction method, label-free biosensors can be generally classified as electrical, optical and mechanical techniques which can usually provide complementary results.

Label-free optical biosensors are principally based on the measurement of refractive index change originated from the presence of analytes on the optical transducer surface. The sensitive waveguide based techniques have presented their performances in several applications, including *in situ* monitoring of the construction of polyelectrolyte multilayers^4–6^, adsorption of biomolecules^7^ as well as monitoring the adhesion of living cells^8^. Beside the simplest and most traditional optical waveguide lightmode spectroscopy (OWLS) technique, the recently introduced waveguide-based instruments, such as resonant waveguide grating (RWG) and grating coupled interferometry (GCI) biosensors have shown the potentials of this measurement principle in terms of throughput and achieving top sensitivities^3,9,10^. Apart from the plentiful applications, an important limitation of these techniques is that the water, “trapped” in the analyte layers, cannot be detected, because all of these devices are monitoring refractive index contrast compared to the aqueous background. This fact means that only the “dry” mass of analyte molecules or living cells can be determined^11,12^.

Quartz crystal microbalance (QCM) is a mechanical label-free biosensor that measures the shift in resonance frequency of the quartz crystal generated by the deposited analyte molecules or cells on its surface. This technique is able to measure the hydrated mass of the examined layers, since the frequency shift is sensitive to the surface-bound water as well. Furthermore, viscoelastic properties of layers can be gained by the *in situ* monitoring of energy dissipation that is usually related to the presence of soft, hydrated films on the sensor surface. QCM has shown its potentials, especially for investigating highly hydrated layers formed by both synthetic polymers or biomaterials^13–16^.

Due to the complexity and nano-scale dimensions of the studied objects on surfaces, complementary analytical techniques afford better insight into the structure and behavior of layers, especially in case of heavily hydrated ultrathin films. The combination of QCM data with results obtained from optical methods is an attractive tool for characterizing the hydration properties of the formed thin films of biopolymers, because measuring the dry and hydrated mass provides quantitative information about the amount of bound water inside the analyte layer^17^. Precedent combined techniques are the simultaneous QCM-SPR^18,19^ and QCM-ellipsometry^20^ measurements, however, only few cases have been presented when the technique can be applied on the same surface enabling simultaneous measurements exactly on the same target objects (ellipsometry compatible QCM module)^21,22^. In spite of the expanding field of combined label-free instruments and the advantages of waveguide based methods over SPR and ellipsometry, so far the realization of an instrumental combination of the OWLS or other waveguide-based methods with a mechanical principle based technique such as QCM is still missing. The combinations of QCM and OWLS data obtained from parallel measurements can be however prosperously applied^11,12,23^.

In the present study, a new compact QCM instrument the so-called QCM-I (QCM with impedance measurement), developed and launched by MicroVacuum Ltd. (Budapest, Hungary), was used to interrogate QCM chips. Compared to the conventional setups, QCM-I applies a different method for obtaining simultaneous frequency and dissipation data. Dissipation can be measured by two types of read-out techniques in QCM devices^24^: (*i*) using impulse excitation method (also called as ring-down method) with the measurement of the decay of crystal’s oscillation after the excitation has been turned off, or (*ii*) by impedance analysis using a network analyzer that measures the frequency spectrum of impedance. The widely used QCM instruments with the trademark of QCM-D (QCM with dissipation monitoring), commercialized by QSense (Biolin Scientific, Sweden) apply the ring-down method^25^.

Numerous ultrathin interface layers have been already introduced for practical applications, however, many of their structure and properties are shallowly explored due to the lack of techniques which would be able to allow broad insight, especially when the measurement is conducted in the presence of solvent, e.g. in aqueous environments. Ultrathin films constructed of highly hydrated polymers such as dextran or poly(L-lysine)-*graft*-poly(ethylene glycol) (PLL-*g*-PEG) are good examples for layers which firmly represents the difficulties in their analysis. The glucose based biopolymer dextran^26–29^ and the synthetic polymer PLL-*g*-PEG^30,31^ are used as coating materials in label-free biosensors, primarily for reducing the non-specific binding, the dominating issue of protein adsorption when measurements are conducted in complex matrices. The advanced hydrophilicity of these polymer chains is supposed to have a principal role in the protein resistant ability^32,33^. Due to the hydrophilic characteristic, the formation of dextran and PLL-*g*-PEG layers accompanies with a high water enrichment in the layer, as a large amount of water can be bound and trapped by the polymer chains^27^. Whilst PLL-*g*-PEG layers are stabilized by electrostatic interactions between the negatively charged oxide surfaces and positively charged PLL backbone^30^, dextran coatings are generally fabricated using privileging methods for covalent grafting^26,34,35^. Instead of native dextran, many applications prefer carboxymethyl dextran (CMD) due to the ease of conjugation with receptor molecules and chemical possibility to attach the polymer chains covalently to transducer surfaces. One possible way to graft CMD to silicon-oxide type surfaces is the reaction of amino groups of the aminosilylated surface with CMD, activated by carbodiimide and succinimide reagents on its carboxyl moieties in order to generate amide bonds^36,37^. This “grafting to” method yields relatively low-density CMD chains on the surface with mostly trains and few loops among the possible chain conformations. In our previous study^37^, we first explored the optical anisotropy of such covalently grafted CMD chains, by measurements using the *in situ* OWLS technique. As we presented in that paper, an overestimated refractive index of the CMD layer was measured owing to the prevalently lain down chain conformation which accompanied with an underestimated value in the calculated layer thickness as well. We proposed QCM-I to be a promising method to clarify the realistic thickness of the CMD layer, to determine its hydration degree and viscoelastic properties, as well as to analyze the anisotropic characteristic and chain conformation in terms of hydration behavior. As the best of our knowledge, OWLS and QCM data have never been combined for time-dependent optogeometric analysis as a function of hydration or viscoelasticity, potentially revealing chain conformations and reorientations. Despite the CMD is a common material in biosensor applications, viscoelastic analysis of covalently grafted ultrathin CMD films is also missing from the available publications and the hydration properties and the time-dependent structure of these layers are still not deeply understood.

**Figure 1:**
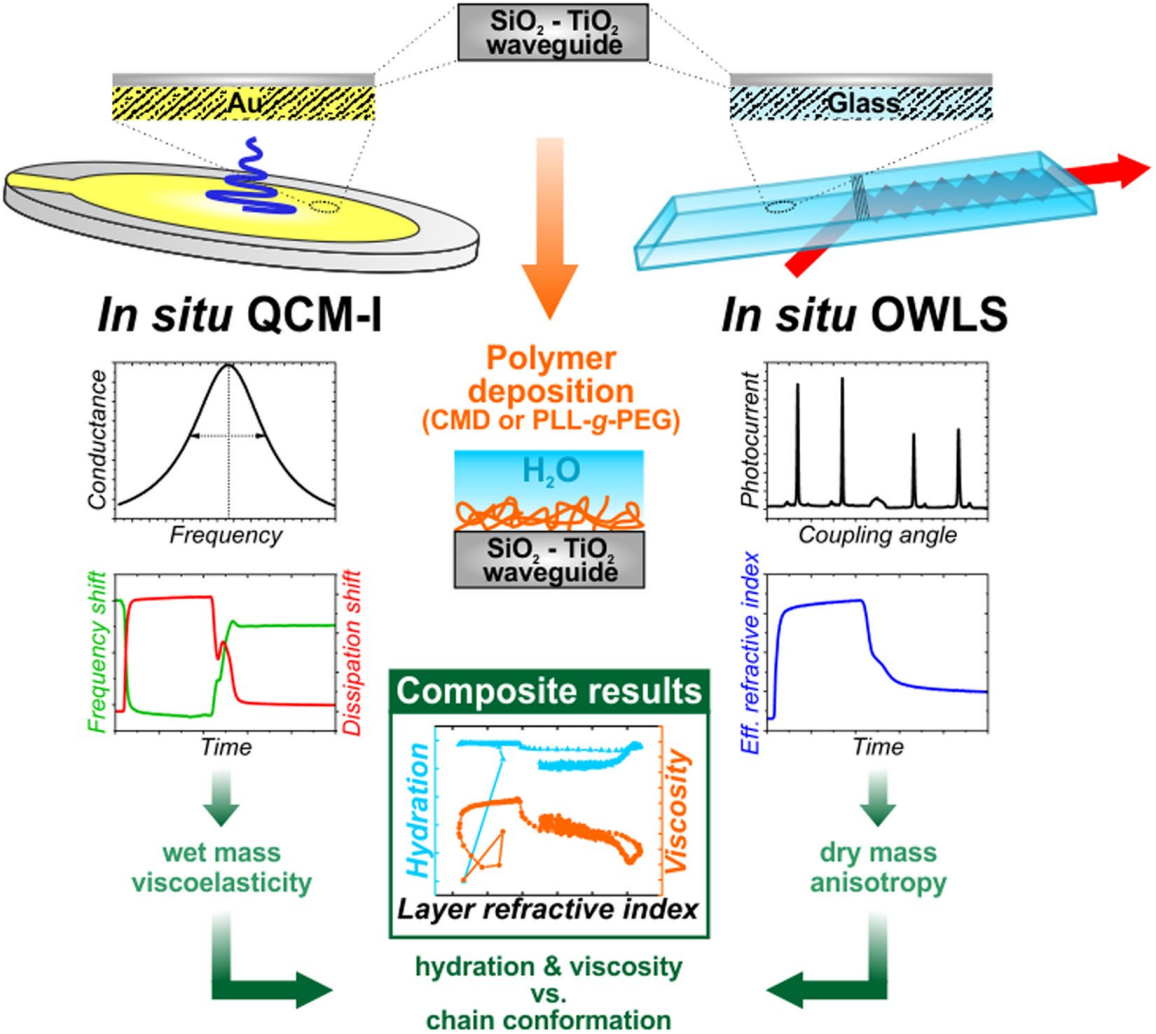
Schematic representation of the measurement and data evaluation methodology developed and applied in this work.

In the present work, we introduce a recently developed QCM-I instrument fitted to parallel OWLS measurements by employing specially designed QCM-I chips with a film of SiO_2_-TiO_2_ waveguide material on top of the gold layer. These developments are able to open the way to parallel OWLS and QCM measurements on exactly the same type of surfaces. In order to demonstrate its capabilities, the formation of CMD and PLL-*g*-PEG layers on the surface was measured *in situ* and the obtained kinetic QCM-I data were analyzed by our MATLAB code. The mass values obtained from the *in situ* QCM-I experiments were supported by *in situ* OWLS data from parallel experiments. The applied methodology allowed to determine and compare the hydration degree of the ultrathin, physically adsorbed PLL-*g*-PEG and covalently grafted CMD layers. In addition to this conventional data analysis method, we present a novel protocol that we developed in order to simultaneously characterize chain conformation and hydration at different layer formation phases and provide a general tool for achieving new insight into the behavior of heavily hydrated ultrathin layers. Using this method, we could make further deductions for the developed CMD layers in terms of dominating conformation of hydrated chains (the applied methodology is schematically represented in Fig. 1). Whilst such examinations are rare and insufficient in the literature for CMD layers, more data are available for the PLL-*g*-PEG (e.g.: deposited mass, viscoelastic and hydration properties). Our measurements on PLL-*g*-PEG layers were able to provide adequate basis for validating the data obtained from measurements with the QCM-I device and verifying our results on CMD films which have not been reported so far.

## Results

### Viscoelastic properties of CMD and PLL-*g*-PEG layers

There are some simple data representation methods which are useful to demonstrate how adequately the Sauerbrey equation can be applied for calculating the mass of a specific layer. At the first glance, the comparison of Δ*f*_*n*_/*n*, the values of the normalized frequency shift at given *n* overtones can be rather suggestive^38^, because a significant dependence of Δ*f*_*n*_/*n* on the overtone number implies a viscoelastic case. Additionally, the magnitude of dissipation shift at the examined *n* overtones (Δ*D*_*n*_) can be also a good indicator. Critical number of 2 × 10^−6^ for dissipation shift has been proposed as the upper limit for treating a layer as rigid^39,40^.

**Figure 2:**
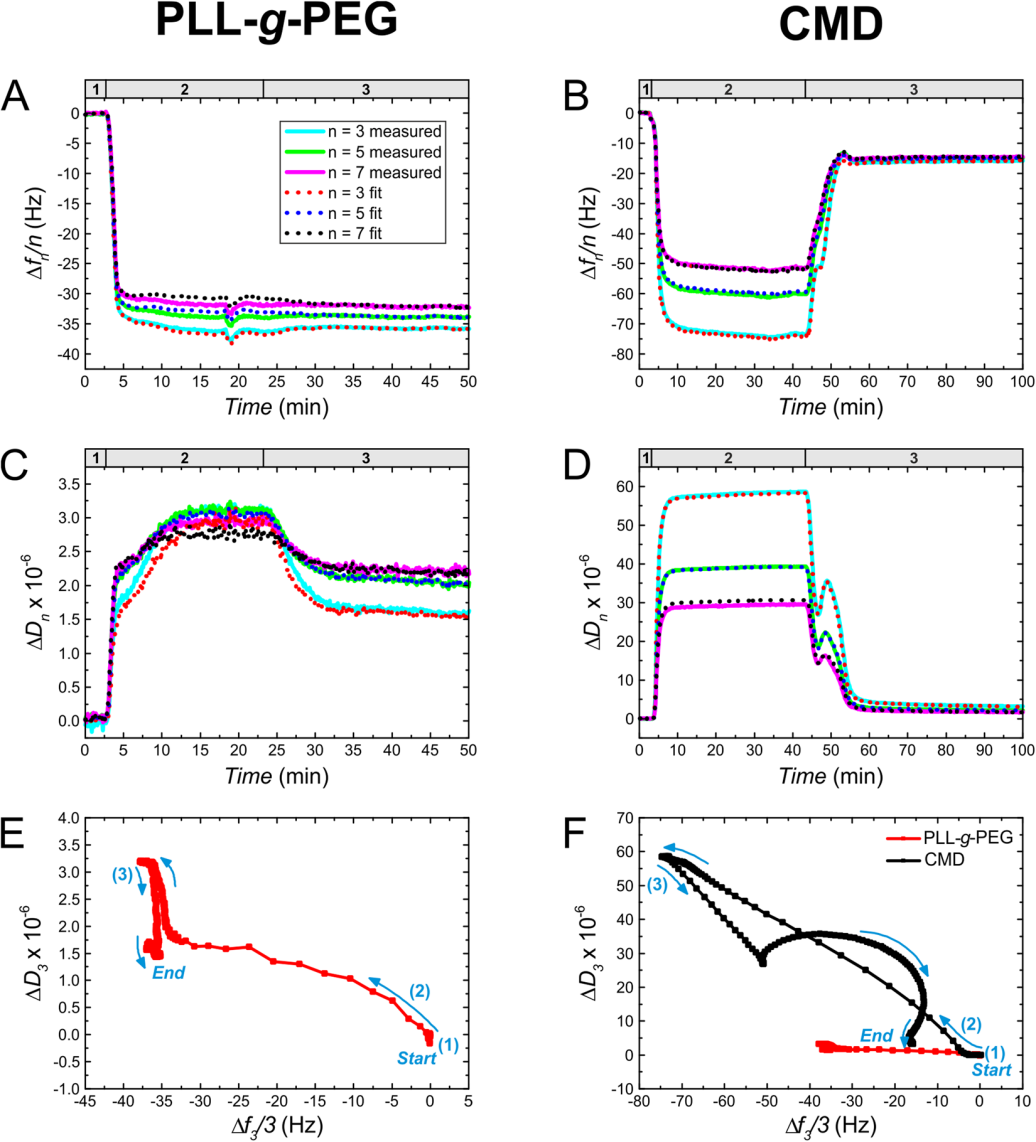
Measured frequency and dissipation shifts as well as corresponding model fits **A**, **B**, **C**, **D**. Normalized frequency (**A**,**B**) and dissipation (**C**,**D**) shift curves measured as a function of time by *in situ* QCM-I on the deposition of PLL-*g*-PEG (**A**,**C**) and CMD layers (**B**,**D**) onto SiO_2_-TiO_2_ surfaces. The data are plotted for the overtones *n* = 3, 5, 7 (see the legend in graph **A**, which is also related to graph **B**,**C** and **D**). The experiments were performed in three consecutive steps: the polymer-free solution was first driven through the flow-cell to have a stable baseline (1); then the solvent was exchanged to the polymer solution (2), which was flowed until it was replaced by the solvent (3) in order to wash away the loosely adsorbed polymer chains. The model fits of the measured normalized frequency and dissipation shifts for the overtones 3, 5 and 7 are also shown. We quantified the quality of fit for the entire fitted time interval by the mean squared error (MSE) number. In case of the shown fits, MSE = 117 and 22 could be reached for PLL-*g*-PEG and CMD measurements, respectively. (**E**,**F**) Dissipation shift against normalized frequency shift curves for PLL-*g*-PEG (**E**) and CMD (**F**). The arrows show the direction of the experiment in time, while the numbers indicate the specific experimental sections as explained above (1: baseline, 2: deposition, 3: washing). The red curve in plot **E** is enlarged from plot **F**.

Table 1 summarizes the Δ*f*_*n*_/*n* and Δ*D*_*n*_ values for the irreversibly attached layers. Due to the sensitivity of fundamental frequency (*n* = 1) to the environmental noise^38^, fundamental resonance parameters were usually neglected and Δ*f*, Δ*D* data only for the overtones of *n* > 1 were presented. The data were read at the end point of the corresponding curves in graph **A** and **B** of Fig. 2. The measured Δ*f*_*n*_/*n* values followed only a small dependence on the overtone number, usually suggesting that the Sauerbrey equation could be rather adequate for calculating the deposited mass of PLL-*g*-PEG and CMD layers and therefore these layers were supposed to be rigid. In contrast to this observation, the values of Δ*D*_*n*_ (>2 × 10^−6^) indicated significant viscoelastic behavior that was particularly expressed in case of CMD. For PLL-*g*-PEG, Δ*D*_*n*_ values (≈2 × 10^−6^) were around the proposed boundary^39,40^.

**Table 1:** Normalized frequency and dissipation shifts for PLL-*g*-PEG and CMD layers at the overtones of *n* = 3, 5, 7.

		PLL-*g*-PEG	CMD
Δ*f*_*n*_*/n* (Hz)	*n* = 3	−32.5 ± 3.7	−15.7 ± 1.6
	*n* = 5	−30.6 ± 4.3	−14.7 ± 1.6
	*n* = 7	−29.2 ± 4.8	−14.3 ± 1.9
Δ*D*_*n*_ × 10^−6^	*n* = 3	1.80 ± 0.95	3.68 ± 1.46
	*n* = 5	2.12 ± 0.90	2.28 ± 0.79
	*n* = 7	2.17 ± 0.68	1.73 ± 0.49

The data are presented as averaged values ± standard deviation, calculated from 3 parallel experiments both for PLL-*g*-PEG and CMD.

*In situ* QCM-I measurements enabled to investigate not only the properties of the remained layer at the end of the experiment, but also the progress of layer formation. Comparing the obtained Δ*f*_*n*_*/n* and Δ*D*_*n*_ graphs of CMD and PLL-*g*-PEG in Fig. 2, the data indicate considerable differences in the behavior of the two layers. While most of the adsorbed PLL-*g*-PEG was irreversibly attached to the surface (graph **A**, small change in Δ*f*_*n*_*/n* as a function of time), a significant amount of CMD could be washed off (**B**). It is also clear from graphs **C** and **D**, that the viscoelastic properties were strongly affected by rinsing the layer, demonstrated indirectly by Δ*D* in time. The relatively high shift in Δ*D* of PLL-*g*-PEG against the unwashed state suggests that peeling of a small number of weakly bound chains could lead to a perceivable alteration in the rigidity of the layer. The effect of rinsing was much more notable in case of CMD. Despite the frequency shift caused by the formed CMD layer was more than two-folds compared to PLL-*g*-PEG, about 80% of the frequency shift was retrieved by desorbing during the washing and the dissipation of the remained layer turned to Δ*D*_3_ = 4.8 × 10^−6^ from a strongly dissipative state (Δ*D*_3_ ≈ 60 × 10^−6^ compared to the value of 2.5 × 10^−6^ for PLL-*g*-PEG). Therefore, it is supposed that the CMD layer consisted of mostly loosely bound chains during the deposition phase, providing a soft coverage. Afterwards, these chains were easily desorbed, and a thin covalently grafted film could only remain.

Graphs **E** and **F** clearly illustrate and confirm the effect of washing, where one can track the change of Δ*D* against Δ*f* in the whole experimental timescale. The significant bend in the CMD curve (graph **F**) originates from the peak in Δ*f* and Δ*D* during the washing section when intense desorption of the loosely attached chains was in progress. The analysis of this observation supported by OWLS data will be demonstrated in the subsequent part of the Discussion (Section 2.3).

Since the relatively high value of Δ*D* suggested a viscoelastic case, we used our evaluation code to determine the hydrated mass (*M*_A_^QCM,V^, where *V* in the superscript refers to the Voigt-based model), shear viscosity (*η*_A_) as well as shear elastic modulus (*µ*_A_) of the adlayers. Beside the measured Δ*f* and Δ*D* data, the Voigt-based viscoelastic model requires the effective density of hydrated adlayer (*ρ*_A_) as an input parameter. In case of heavily hydrated layers, *ρ*_A_ can be approximated with the density of water, thus, *ρ*_A_ = 1000 kg/m^3^ was used both for CMD and PLL-*g*-PEG^23^.

The results computed by fitting the measured Δ*f* and Δ*D* data are presented in Table 2 (data were given for the end point of the experiment, representing the remained stable layer on the surface), and the obtained fits of measured Δ*f* and Δ*D* data for one PLL-*g*-PEG and one CMD measurement are shown in Fig. 2. Herein, graph **A**, **B**, **C** and **D** represent the fitted curves for the entire experimental timescale together with the corresponding measurements. Note that during the experimental section of polymer deposition, the resulted data must be carefully considered, because the same fixed bulk parameters (viscosity, modulus and density) were used in the model as in the baseline section, however they were actually unknown. This could be especially true in the case of CMD experiments, where the solution with a relatively high concentration (50 mg/ml) was applied.

**Table 2:** Summary and literature review of results obtained from parallel OWLS and QCM-I measurements on PLL-*g*-PEG and CMD layers

	Measured		Reference^a^	
	PLL-*g*-PEG	CMD	PLL-*g*-PEG or PEG^b^	PLL-*g*-D^c^, D^d^, CMC^e^
*M*_A_^OWLS^ (ng/cm^2^)	274 ± 36	124 ± 23	268 ± 14^43^, 198 ± 12^23^	—
*M*_A_^QCM,S3^ (ng/cm^2^)	575 ± 66	277 ± 28	—	—
*M*_A_^QCM,V^ (ng/cm^2^)	618 ± 36	1102 ± 487	690^43^, 1241 ± 63^23^	—
*φ*_A_ (%)	56	89	72^43^, 83^44^, 84^23^	57^43^^(c)^, 60–70^59^^(c)^
${N}_{{H}_{2}O/monomer}$	4	71	7^43^, 14^23^	14^43^^(c)^, 15–30^59^^(c)^
*ρ*_A_ (kg/m^3^)	1000	1000	1000^23^	1000^60^^(d)^
*n*_A_	1.58 ± 0.04	1.66 ± 0.22	—	—
*d*_A_^OWLS^ (nm)	2.1 ± 0.1	0.9 ± 0.5	—	—
*d*_A_^QCM,S3^ (nm)	5.8 ± 0.7	2.8 ± 0.3	—	—
*d*_A_^QCM,V^ (nm)	6.2 ± 0.4	11.0 ± 4.9	5.9^41^, 8.5 ± 0.4^44^	—
*η*_A_ (mPa·s)	3.71 ± 1.22	1.43 ± 0.27	1.7^44^, 1.4^38^^(b)^	0.50 ± 0.26^60^^(d)^
*µ*_A_ (MPa)	0.80 ± 0.46	0.029 ± 0.014	0.10^43^, 0.125^38^^(b)^	0.1–0.2^39^^(e)^

The data are presented as averaged values ± standard deviation calculated from 3 repeated experiments both for PLL-*g*-PEG and CMD as well as for OWLS and QCM-I measurements. The superscripted indications are devoted to set the following remarks: ^a^some referred data were not concretely measured on PLL-*g*-PEG and CMD polymers (see details below); ^b^measured on PEG; ^c^measured on PLL-*g*-D (D as dextran); ^d^measured on dextran; ^e^measured on carboxymethyl cellulose (CMC).

*M*_A_^OWLS^: areal mass density measured by OWLS; *M*_A_^QCM,S3^, *M*_A_^QCM,V^: areal mass density measured by QCM-I and evaluated by the Sauerbrey equation as well as by the Voigt-based model, respectively; *φ*_A_: hydration degree; ${N}_{{H}_{2}O/monomer}$: number of bound water molecules per one monomer unit of the polymer chain; *ρ*_A_: density of the hydrated polymer adlayer; *n*_A_, *d*_A_^OWLS^: refractive index and optical thickness of the adlayer determined by OWLS; *d*_A_^QCM,S3^*, d*_A_^QCM,V^: adlayer thickness evaluated by the Sauerbrey equation as well as by the Voigt-based model, respectively; *η*_A_, *µ*_A_: shear viscosity and shear elastic modulus of the polymer adlayer evaluated by the Voigt-based model.

The references are indicated by the numbers in the superscripts.

**Figure 3:**
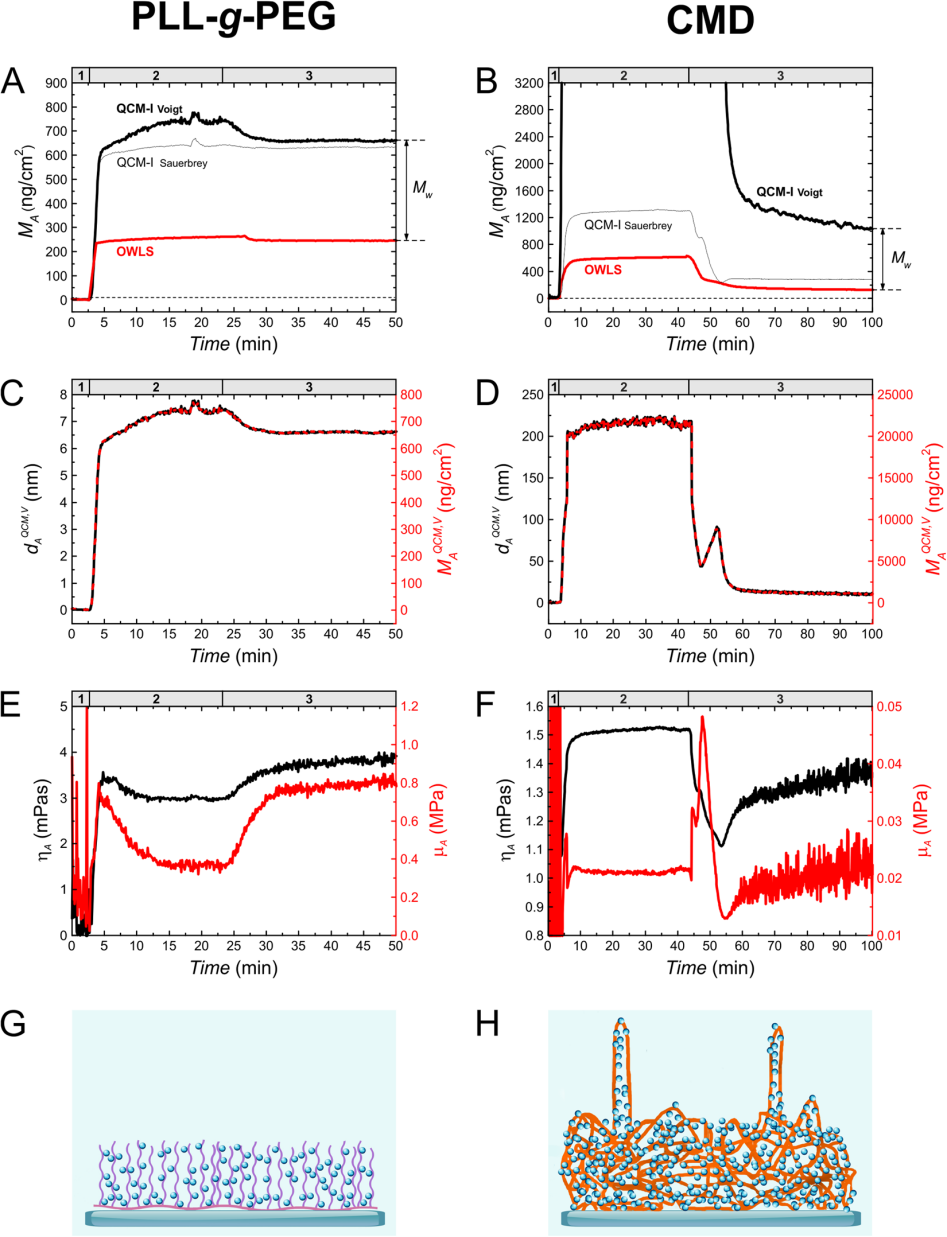
Results from the Voigt-based viscoelastic model and determination of the mass of bound water. (**A**,**B**) Areal mass density sensograms of *in situ* QCM-I (solid black line) and OWLS (solid red line) measurements on PLL-*g*-PEG (**A**) and CMD (**B**) deposition experiments. The numbers in the headers indicate the corresponding experimental sections (detailed in Fig. 2). Two types of QCM-I mass interpretations are plotted. The thicker lines belong to the mass data calculated by the Voigt-based model, while the thinner lines correspond to the 3rd overtone Sauerbrey mass (S3). In case of the CMD plot (**B**), the data of the fitted Voigt mass were smoothed by adjacent-averaging method using a 10-point averaging window. The dashed line underneath is an extension of the baseline. At the deposition section of CMD experiment, the calculated Voigt mass was too high for a proper interpretation, therefore only the masses up to 3200 ng/cm^2^ are shown. (**C**,**D**) Full mass (dashed red) as well as thickness plots (solid black curves) are shown in graph **C** and **D**. The thickness and mass curves have the same shape as they are simply proportional by the factor of layer density (Eq. (5), Section 4.4). The layer density was fixed at 1000 kg/m^3^. (**E**,**F**) Additional model calculation results as a function of time: shear viscosity (black) and shear elastic modulus (red) of PLL-*g*-PEG and CMD adlayers. (**G**,**H**) The drawings are demonstrating the proposed structure of the formed polymer layers, where both the polymer chains and bound water molecules are indicated.

The resulted time-dependent surface mass densities are shown in graphs **A** and **B** of Fig. 3 which combine the mass data obtained from OWLS and QCM-I measurements. In addition, graph **C** and **D** represent the thickness as well as **E** and **F** the viscosity and modulus data calculated by the fits. At the bottom, the proposed schematic structures of the attached layers are also depicted (**G**, **H**).

Due to the fact that the measurements with the applied QCM-I instrument have not been reported in the scientific literature before, in order to validate our measured and evaluated data, we compared the results with published ones which were obtained from similar QCM-D experiments or from other reliable techniques. Even for the widely studied PLL-*g*-PEG layers, although mass data are well published, viscoelastic parameters are less available. In the work of Perry *et al*., 75.2 ng/cm^2^ was quantified for the dry mass of PLL-*g*-PEG layer^41^ on silica surface using the OWLS technique. The thickness of those layers in hydrated state was found to be 5.91 nm, which is in good agreement with our findings. In another study, where a PLL-*g*-PEG polymer with higher molecular weight PEG was used (PLL(20)-*g*[3.5]-PEG(5)), higher dry (198 ng/cm^2^) and wet mass (1241 ng/cm^2^) could be observed by OWLS and QCM-D^23^. In order to demonstrate the diversity of the literature values, more reference data are compiled in Table 2. The determined viscoelastic parameters (*η*_A_ and *µ*_A_) were higher than the literature data, which can be attributed to the lower water content of the layer. Our results derived from the viscoelastic analysis were in good agreement with published observations, nevertheless they should be considered carefully due to the limitations of the Voinova’s model^42^.

Since elaborate QCM studies on ultrathin CMD nanolayers are missing in the literature, our data could be only compared with data obtained on similar materials. The reference values for comparison are summarized in Table 2.

### Comparison of hydration degrees

While OWLS measures only the deposited mass of the polymer layer without the bound or trapped solvent molecules (“dry mass”), QCM measures the solvated mass of the polymer chains (“wet mass”). Comparing the mass density data derived from OWLS measurements (*M*_A_^OWLS^) with the QCM mass densities calculated by the fit of the Voigt-based model (*M*_A_^QCM,V^) (Table 2) one can conclude that there is a significant difference between the PLL-*g*-PEG and CMD both in the adsorbed amount of polymer chains (“dry mass”) and in their hydrated state (“wet mass”). The difference between the dry and wet masses are expressively illustrated in Fig. 3 (graphs **A** and **B**), where the amount of bound water (*M*_w_) in the remained layers is also indicated. Using the two obtained mass data, the hydration degree φ_A_ with respect to the wet mass can be calculated as follows^20^:

1 $${\phi }_{A}=\frac{{M}_{A}^{QCM,V}-{M}_{A}^{OWLS}}{{M}_{A}^{QCM,V}}\times 100$$

Both for the PLL-*g*-PEG and CMD layers, high water content could be determined, with values of φ_A_ = 56% and 89%. It is noted that for PLL-*g*-PEG layer a higher hydration of 72–84% can be found in recent reports^23,43,44^. While the dry mass of the polymer chains was lower for the CMD, its hydrated mass significantly exceeded the hydrated mass of PLL-*g*-PEG, suggesting the prominent hydration ability of CMD molecules. The huge difference between the Sauerbrey (*M*_A_^QCM,S3^) and Voigt CMD mass clearly verify the application of the viscoelastic model in the data analysis. The number of bound water molecules per one monomer unit of the polymer chain (${N}_{{H}_{2}O/monomer}$) was also calculated according to the method of Müller and co-workers^23^ (see Table 2).

### Kinetic analysis of CMD deposition – conformational changes of polymer chains in terms of hydration and viscoelasticity

To reveal time-dependent changes in the formation of heavily hydrated CMD nanolayers, we developed a novel OWLS-QCM data analyzing methodology based on the quasi-isotropic analysis of OWLS data combined with derivative quantities (hydration degree, viscosity) gained from parallel QCM-I measurements. The conventional quasi-isotropic analysis of optogeometric results calculated from OWLS data using the 4-layer homogenous isotropic mode equations was introduced by Horvath and Ramsden^45^, however it has never been applied in combination with QCM data. It was shown that the sign of the deviation between the calculated refractive index (*n*_A_) and expected (realistic) refractive index of adlayer suggests a positive or negative birefringence that can be also expressed by the relation of the ordinary (*n*_A,o_) and extraordinary (*n*_A,e_) components of *n*_A_. According to these considerations, if the determined *n*_A_ underestimates the expected layer refractive index, the chains are supposed to pose dominantly perpendicular to the surface which is a case of positive birefringence (*n*_A,o_ < *n*_A,e_). In case of an overestimated *n*_A_, the prevalent chain conformation is implied to be parallel to the surface (negative birefringence, *n*_A,o_ > *n*_A,e_).

**Figure 4:**
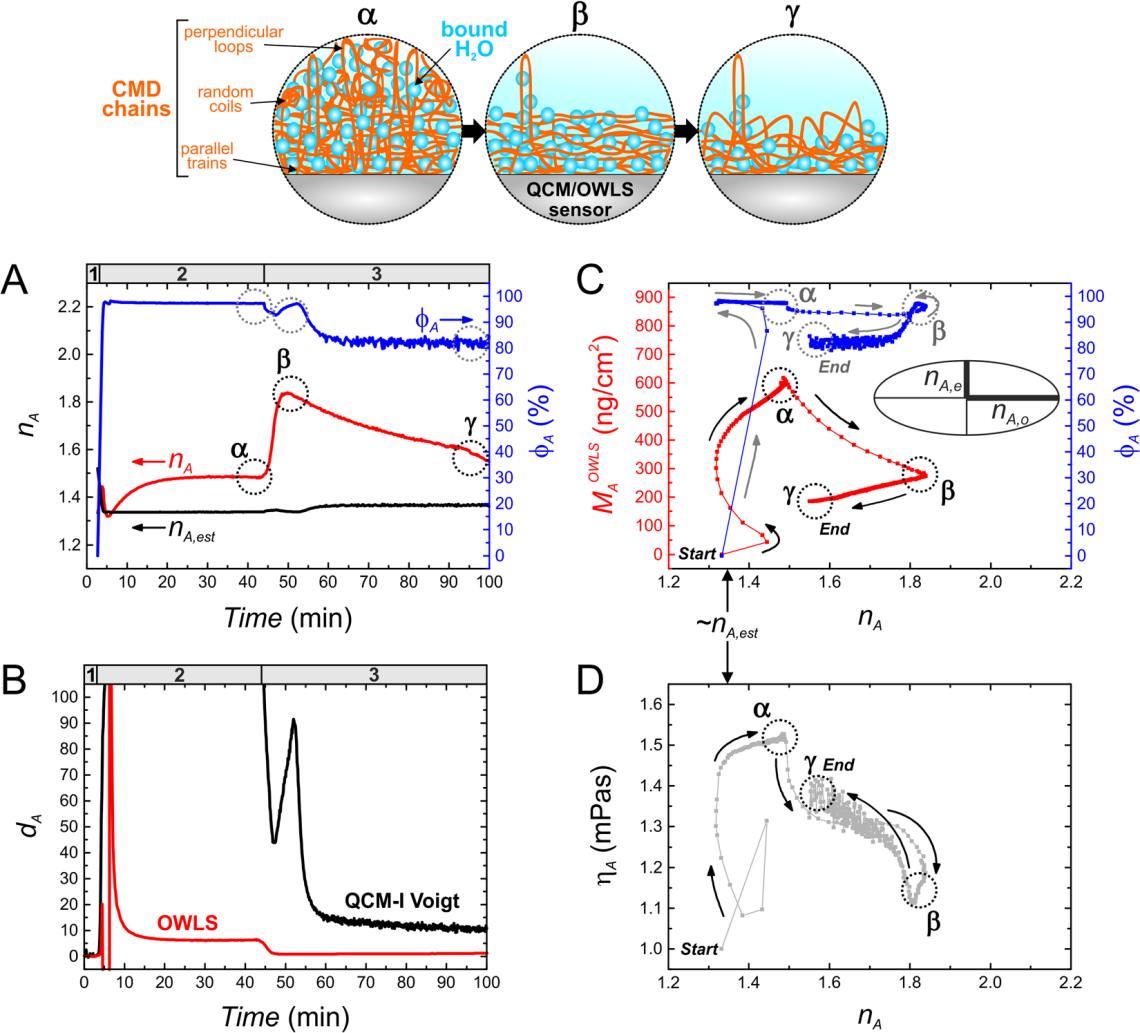
Revealed conformational changes in CMD layers in terms of hydration and viscoelastic properties, based on our developed data analysis methodology and the resulted composite graphs. The illustrations on the top demonstrate the assumed conformational changes in the formed CMD layer, relating to certain experimental phases (times) which are indicated in the corresponding graphs by **α**, **β** and **γ**, respectively. The numbers in the headers indicate the experimental sections which are detailed in Fig. 2. Graph **A** and **B** represent the adlayer refractive index (*n*_A_) and hydration (*φ*_A_) as well as mechanical and optical thickness data (*d*_A_^OWLS^ and *d*_A_^QCM,V^) obtained at the whole experimental timescale. Graph **B** shows the thicknesses originating from both the OWLS and QCM-I measurements, using the same hydrated thickness data of graph **D** in Fig. 3. The values of *n*_A_ and *d*_A_ were calculated from OWLS measurements using the isotropic homogenous 4-layer mode equations. The estimated (realistic) refractive index of the CMD layer (*n*_A,est_) was also calculated, based on the known refractive index of bulk dextran and determined hydration degree. These *n*_A,est_ values (1.33–1.36) provided a practical boundary for the isotropic-anisotropic transition (shown in graph **A**,**C** and **D**). Graph **C** was compiled from *n*_A_ and *φ*_A_ data of graph **A**, providing useful physical parameters over the measured dry CMD masses (*M*_A_^OWLS^). In the inset of graph **C**, the refractive index ellipsoid demonstrates the observed negative birefringence in the adlayer (*n*_A,o_ > *n*_A,e_). Graph **D** combines the magnitude of anisotropy (*n*_A_) with layer viscosity (*η*_A_), where the latter quantity was independently determined from QCM-I.

Graph **A** of Fig. 4 presents both the *n*_A_ and *φ*_A_ parameters of CMD layer at the experimental timescale. The refractive index of the bulk dextran^29^ (plausible approximation for the refractive index of CMD) and the determined *φ*_A_ allowed to estimate the time-dependent values of the CMD layer’s realistic refractive index (*n*_A,est_) and to present its time dependence. It was obvious that the applied 4-layer mode equation model provided unrealistic *n*_A_ for the whole experiment. Moreover, since the magnitude of this overestimation correlates with the extent of anisotropy^45^, the time-dependent changes in the value of *n*_A_ allowed to deduce structural changes of the CMD layer. The overestimation of *n*_A_ is also expressed in the underestimated values of optical thickness (*d*_A_^OWLS^) originated from OWLS data as well. In Fig. 4, graph **B** demonstrates the differences to the realistic hydrated thickness obtained from QCM-I measurements.

For the better understanding of conformational alterations, graph **C** and **D** show the curves of the dry CMD mass (*M*_A_^OWLS^) combined with *φ*_A_ as a function of *n*_A_ as well as *η*_A_ as a function of *n*_A_, respectively. Owing to this special data interpretation that involves time as an implicit parameter, one can track the effect of polymer deposition as well as the effect of washing on the layer structure. Based on the data, the inset figures **α**, **β** and **γ** illustrate the devised layer structures, relating to certain phases of the layer fabrication timescale. The formation and desorption of CMD layer is presented as follows. Right after the CMD solution inflow, a thick, heavily hydrated (*φ*_A_ ≈ 97%) and viscous (*η*_A_ ≈ 1.52 mPa∙s) layer was formed and stabilized in which the dominant chain conformation was parallel to the surface (**α**). The increased magnitude of anisotropy (*n*_A_ = 1.49 vs. *n*_A,est_ = 1.34) suggests only few perpendicular loops and attached random coils in the layer. However, in the forthcoming washing phase, a huge and fast alteration occurred in the layer structure (*n*_A_ = 1.49 → 1.84) that was induced by the removal of loosely bound chains mostly with perpendicular and random coil conformations. As a result, the remaining layer (at ca. 50 min, indicated by **β**) consisted of prevalently lain down chains (so-called train conformation) with only very few extending loops.

Nevertheless, an unexpected bend appeared around phase **β** in the curve of *φ*_A_ (see graph **C**, transiently increasing *φ*_A_ values). In addition, **β** was followed by a continuous decrease in *n*_A_ and increase in *η*_A_ (**D**) presenting an opposite direction to the preceding tendency. The bend corresponds to the indicated peak around **β** in the time-dependent *φ*_A_ curve of graph **A** and it could be originated from the peak appeared in the Δ*D*_*n*_ and Δ*f*_*n*_/*n* curves as well as from the bend in the Δ*D*_3_ − Δ*f*_*3*_/3 curve, respectively (Fig. 2**B**,**D**,**F**). Whilst the phenomenon around **β** could not be perceived by the raw effective refractive index and calculated OWLS mass data, it was clearly revealed by the alteration of *n*_A_. Based on the above considerations, we strongly argue that after the rapid displacement of weakly bound CMD molecules, a conformational rearrangement started in the layer structure, accompanying with a moderate loss of mass and involving the buckling of lain down chains to extending loops (descending *n*_A_). The rearrangement was accompanied with a transient rehydration of the layer (see the small bend in the *φ*_A_ curve, graph **C**), followed by a recommencing dehydration that should have been governed by the slight detachment of CMD chains (moderate change in *M*_A_^OWLS^ after **β**). The presence of chains with higher extension and less hydration is also backed up by the observed rise in layer viscosity (graph **D**).

The fact that the massive decreasing tendency of *n*_A_ could be still observed when the surface amount of both the CMD chains and trapped water was stabilized (only small level of mass loss), strongly confirms the conformational rearrangement hypothesis. It is also concluded that despite the emergence of perpendicularly oriented loops, the lain down chains were still dominating in the remained CMD layer structure (**γ**), as demonstrated by the relatively high *n*_A_ values (1.55 > *n*_A,est_ = 1.36, significant negative birefringence).

As it is supported by all the analyzed data (*n*_A_, *d*_A_, *M*_A_^OWLS^, *φ*_A_, *η*_A_) and due to the fact that separate effects of the conformational rearrangement could be independently detected by OWLS and QCM-I measurements, the presented layer formation mechanism is based on plausible considerations. It is worth highlighting that without the quasi-isotropic analysis, the conformational rearrangement could not have been observed by OWLS (the raw QCM-I data were yet sensitive). Our present results coincide well with our previous findings on the conformational alterations in ultrathin CMD layers^37^. Here we also note that the background of the observed phenomena should be further analyzed due to the complexity (hydrated state, covalent attachment) and nano-scaled dimensions of the examined layers.

## Conclusion

In this study, a newly developed QCM-I instrument based on the impedance measurement read-out method has been presented. The QCM-I chips covered by a film of SiO_2_-TiO_2_ waveguide material provided the ability to have the identical surface chemistry for performing complementary experiments along with the OWLS technique. Electrostatically adsorbed PLL-*g*-PEG and covalently grafted CMD interface layers were deposited and monitored *in situ* by the two biosensor techniques. The well-documented PLL-*g*-PEG was used as a reference system to demonstrate the new QCM-I measurement technique and the developed evaluation methodologies. The results on PLL-*g*-PEG coincide well with the reference data from the literature based on QCM-D measurements, validating the instrument and our QCM-I data evaluation code. Our results on the viscosity, modulus, hydration degree and wet thickness of ultrathin CMD layers have not been published so far to the best of our knowledge. The measurements revealed significant difference between the two polymer nanolayers: the deposited dry mass showed less amount of CMD grafted to the surface than in case of the electrostatically deposited PLL-*g*-PEG. However, the water content of the CMD and PLL-*g*-PEG layer with respect to the solvated mass (hydration degree) was determined to be 89% and 59% revealing a significantly higher hydration capability of the CMD layer. Our evaluation code could be successfully used for viscoelastic analysis: the shear viscosity and shear elastic modulus of the polymer adlayers were determined. The measured data were thoroughly compared with literature values, which can help further studies in layer optimization for biosensing. We developed a novel OWLS-QCM data analysis methodology in order to enlighten the time-dependent structure of CMD nanolayers in terms of hydration and viscoelastic behavior. As a basis, we extended the quasi-anisotropic analysis methodology with the support of QCM-I data. Analyzing the layer formation mechanism, we found that the washing phase induced significant conformational rearrangement in the layer structure resulting in dominantly lain down chains complemented with a moderate number of loop type conformations. The presented results were established by the combined evaluation of anisotropic characteristic, dry mass, hydration degree and layer viscosity.

We successfully demonstrated the ability of our developed analysis protocol that is a useful approach for future investigations of hydrated ultrathin layers based on parallel *in situ* OWLS-QCM measurements.

## Methods

### Chemicals

The synthetic copolymers, poly(L-lysine)-*graft*-poly(ethylene glycol) (PLL-*g*-PEG, with the architecture of PLL(20)-*g*[3.5]-PEG(2), where the molecular weight of the PLL backbone and the PEG chains were 20 kDa and 2 kDa, respectively, and the grafting density [(lysine-mers)/(PEG side chains)] was 3.5) were obtained in its powder form from SuSoS AG (Dübendorf, Switzerland). 4-(2-hydroxyethyl)-1-piperazineethanesulfonic acid (HEPES) was purchased from Sigma-Aldrich. Dextran T-500 (with 500 kDa molecular weight) was obtained from Pharmacosmos A/S (Holbaek, Denmark). 3-aminopropyltriethoxysilane (hereafter aminosilane), Ethyl-3-(3-dimethylaminopropyl)-carbodiimide hydrochloride (EDC) and N-hydroxysuccinimide (NHS) were obtained from Sigma-Aldrich, monochloroacetic acid was purchased from Thermo Fisher. Water used in the experiments was ultrapure grade Milli-Q water with resistivity of 18 MΩ·cm. All chemicals and reagents were of analytical grade.

### Sample preparation

#### Sensor chip cleaning

OWLS chips were cleaned by chromic acid followed by intensive washing with ultrapure water and drying under N_2_ stream. QCM-I chips were first washed by ultrapure water and 10 min exposure to UV ozone cleaner (MicroVacuum Ltd., Budapest, Hungary) was subsequently used.

#### Fabrication of CMD layers

The synthesis of CMD from 500 kDa molecular weight native dextran derived from a published procedure of Huynh *et al*.^46^ and it was detailed in our previous work^37^. The sample preparation for CMD grafting and the grafting process itself was also described in our publication^37^, however, some modifications were implemented. Cleaned chips surfaces were silylated by 3-aminopropyltriethoxysilane in a heated vacuum chamber (Glass oven B-585, BÜCHI Labortechnik AG, Flawil, Switzerland). 200 µl of the aminosilane was placed into a glass vial that was mounted behind the sample rack in the vacuum chamber. Followed by a 10 min incubation at 80 °C under vacuum (≤5 Hg mm), the samples were held without heating for 20 min, and then they were baked at 120 °C for 2 hours in vacuum. The produced aminated surfaces were kept in vacuum desiccator until used.

The CMD used in this work was characterized by a degree of substitution (DS) of 0.6. The CMD solutions were prepared by dissolving lyophilized CMD in water. The pH of the CMD solution was subsequently adjusted by NaOH to pH 7.0–7.2 and the solution was syringe-filtered on 0.2 µm pore size membrane. NHS and EDC were added to the CMD solution and this so-called grafting solution (CMD-EDC-NHS solution) was used after 15 min of incubation.

The grafting procedure of CMD was performed in particular flow-cells of the related QCM-I and OWLS setups, while the surface deposition was monitored *in situ*. The flow rate was maintained at 1 µl/s by peristaltic pump (Ismatec Reglo, Cole-Parmer GmbH, Wertheim, Germany) throughout the experiment. The experiment started with water flow. After reaching a stable baseline, the water was replaced by the CMD-EDC-NHS solution, which was flowed through the cell for 40 min. Afterwards, water was used again for washing the surface until a constant signal could be recorded.

#### Fabrication of PLL-*g*-PEG layers

PLL-*g*-PEG solutions with concentration of 1 mg/ml were prepared in HEPES buffer (with HEPES concentration of 10 mM). The pH of the HEPES was adjusted by NaOH solution to 7.4 before the preparation of the PLL-*g*-PEG solution. The sensor chip surfaces were not chemically modified, they were used in the experiments after the cleaning process. Followed by reaching a stable baseline with HEPES that was also driven by a peristaltic pump (flow rate was 1 µl/s throughout the experiment), the PLL-*g*-PEG solutions were flowed over the sensor chip for 20 min. The washing section was performed with HEPES flow until a constant signal was reached.

### Measurement techniques

#### Optical waveguide lightmode spectroscopy (OWLS)

Optical waveguide lightmode spectroscopy measurements were performed by an ASI BIOS-1 and OWLS 210 instruments (MicroVacuum Ltd., Budapest, Hungary). The optical sensor chips consist of a SiO_2_-TiO_2_ waveguide layer on top of an AF45 glass substrate. The 2400 μV and OW2400 type sensor chips were obtained from MicroVacuum Ltd. The linearly polarized 632.8 nm wavelength He-Ne laser beam was coupled into the waveguide layer by the grating imprinted on the sensor chip. *In situ* measurements were conducted by assembling the chip with a flow-cell, which was set in the goniometer of the instrument. The liquid was flowed over the waveguide surface at a 1 µl/s flow rate by employing a peristaltic pump (Ismatec, Reglo). The coupling angle was scanned with 14 s time resolution.

The refractive indices of the solutions (*n*_c_) were measured separately by a precision refractometer at the wavelength of 632.8 nm (J157 Automatic refractometer, Rudolph Research Analytical). The recorded OWLS data were evaluated by the 4 layer mode equations^47^ and the surface mass density was calculated using the de Feijter’s formula^48^. (Data for the calculations: *dn/dc*_CMD_ = 0.15 cm^3^/g, *dn/dc*_PLL-*g*-PEG_ = 0.139 cm^3^/g, *n*_HEPES_ = 1.332, *n*_water_ = 1.3317; where *dn/dc* is the refractive index increment of the solution of the applied polymer, *n* is the refractive index of the solution indicated by the given subscript. All refractive index data belong to the wavelength of 632.8 nm).

#### Quartz crystal microbalance with impedance measurement (QCM-I)

**Figure 5:**
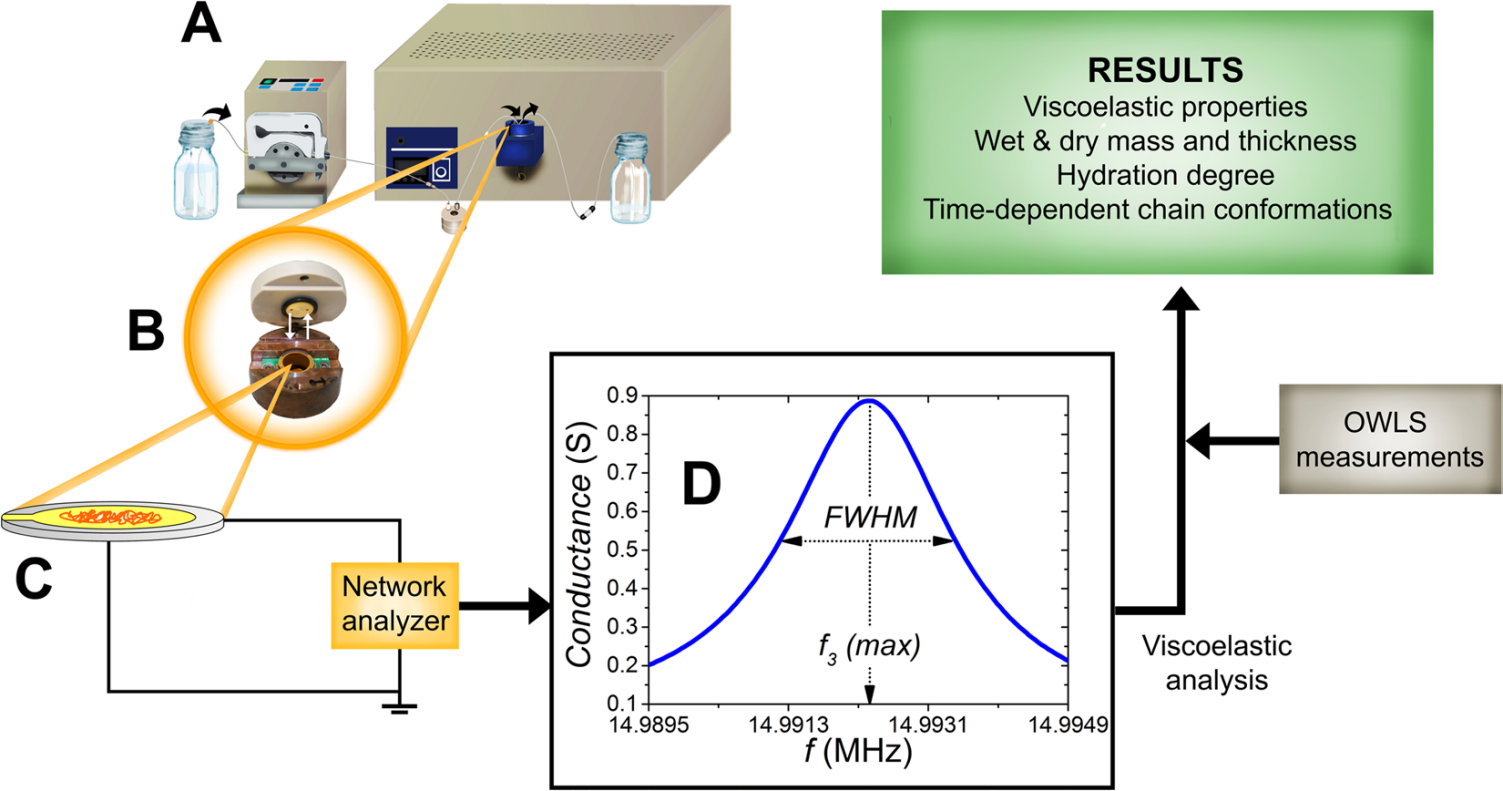
QCM-I measurement setup (**A**) and data evaluation methodology. PLL-*g*-PEG or CMD solutions were flowed into the flow-cell (enlarged in image **B**), where the solution could be facing to the surface of the sensor chip (**C**). Applying a network analyzer, QCM-I measures the frequency spectrum of the conductance (impedance). An obtained resonance peak can be seen around the third overtone resonance frequency (*f*_3_) in graph **D**. Two parameters are quantified by fitting the data points: full width at half maximum (FWHM), proportional to the dissipation as well as resonance frequency at the peak maximum (*f*_3_(max) = *f*_3_). The impedance analysis is performed for each overtone at each measurement time and the *in situ* data provide the basis of the calculations on surface mass density of the interface layer as well as its viscoelastic properties. Complementing the QCM-I results with mass data obtained from OWLS measurements was the basis of determining the hydration degree of the examined polymer layers.

The applied QCM setup was a QCM-I instrument from MicroVacuum Ltd. (Budapest, Hungary)^49^. The resonance sensitivity of the instrument in liquid is 2 × 10^−1^ Hz, the dissipation sensitivity is 1 × 10^−7^ and the mass sensitivity is ≤1 ng/cm^2^, respectively. The flow-cell volume is ~40 µl. The QCM-I sensor chips used in this work were AT-cut crystals with a diameter of 14 mm and a fundamental resonance frequency of 5 MHz. The fabrication of the SiO_2_-TiO_2_ layer on top of the gold is based on the sol-gel method used for the OWLS sensors chip fabrication, and it is prepared in the same composition. The *in situ* experiments were performed at 1 µl/s flow rate maintained by a peristaltic pump. The resonance frequency and dissipation shifts were recorded with the time resolution of 6.2 s for each selected overtone (the overtone numbers were *n* = 1, 3, 5, 7 for the frequencies of 5, 15, 25, 35 MHz, respectively). For the development of the evaluation code, MATLAB® (MathWorks, Inc., Natick, USA) was used as programming environment. The employed QCM-I setup and the methodology of data analysis are illustrated in Fig. 5.

### Viscoelastic analysis of QCM data – implementation in MATLAB

In the widely used QCM-D technique, the amplitude of the recorded oscillation decay (measured electric current as a function of time) is fitted using the parameters of decay time (*τ*) and frequency (*f*) which provide the dimensionless dissipation (*D*) as follows:

2 $$D=\frac{1}{Q}=\frac{1}{\pi f\tau }=\frac{{E}_{dissipated}}{2\pi {E}_{stored}}$$

where *Q* denotes the quality factor, *E*_dissipated_ is the energy dissipated during one oscillatory cycle and *E*_stored_ is the energy stored in the oscillating system. The energy dissipation of the quartz crystal can be obtained as the energy dissipation factor *D* that is the inverse of the frequency quality factor *Q*. The viscoelastic properties of the polymer layers on a QCM crystal can be described by the *D* value^50,51^. The *f* and *D* values measured for multiple overtones allow to deduce the mass and viscoelastic characteristic of the crystal based on continuum mechanical models, most commonly on the Voigt-Kelvin model developed by Voinova and co-workers^52^.

In contrast to the QCM-D, QCM-I applies the impedance analysis as read-out technique. In this case, impedance spectra are recorded for fundamental and overtone frequencies and these spectra are fitted by resonance curves with FWHM (full width at half maximum) and *f* parameters. As it has been shown by the model of electric circuit approach developed by Johannsmann^53^, the formula ΔFWHM/Δ*f* can be associated with the viscoelastic properties of the crystal and its overlayers, thus viscoelastic analysis by the impedance measurement technique is also available. Dissipation can be expressed by the FWHM parameter derived from the impedance analysis:

3 $$D=\frac{1}{Q}=\frac{FWHM}{f}$$

It has been also shown, that the impedance spectrum is retrievable from the oscillation decay by Fourier-transformation^24^. As a result, the two *D* values obtained from the different read-out methods (i.e. QCM-D and QCM-I) can be treated to be equal. Zhang *et al*. pointed out that data recorded by a QCM-D instrument can be accurately evaluated in terms of viscoelastic properties by the equivalent circuit approach^54^, which also works vice versa for the evaluation of data obtained by a QCM-I setup using the Voigt-based model.

The Sauerbrey equation gives a linear relationship between the normalized form of the frequency shift (Δ*f*_*n*_/*n*) and added mass per unit area (*ΔM*_A_^QCM,S^)^55^:

4 $${\rm{\Delta }}{M}_{A}^{QCM,S}=-\,C\frac{{\rm{\Delta }}{f}_{n}}{n}$$

where *C* is the mass sensitivity constant that is dependent only on the physical properties of the quartz crystal, *n* is the overtone number and Δ*f*_*n*_ refers to the frequency shift at a given time *t* (*f(t* = *0)* *−* *f(t)*) at a given *n* overtone. Instead of Δ*M*_A_, hereafter the calculated mass will be indicated by *M*_A_.

Several criteria must be fulfilled for being able to precisely apply the Sauerbrey equation. These criteria demand the examined films to be thin, rigid and evenly distributed on the sensor surface^56^. Especially polymer-based and biological samples tend to be soft and have viscoelastic behavior, causing that their layers cannot entirely follow the motion of the crystal during the QCM measurement. For such layers, the application of the Sauerbrey equation becomes limited, highlighting the need to consider their viscoelastic behavior. A model based on a Voigt viscoelastic element is used to calculate the accurate mass, and additionally, it provides the viscoelastic parameters of the examined layer. This model represents the added viscoelastic layer on the elastic QCM crystal as a viscoelastic element consisted of a spring (elastic part) and dashpot (viscous part). Measuring both the frequency (Δ*f*) and dissipation shifts (Δ*D* = *D(t* = *0)* − *D(t)*) as a function of time enables the quantitative *in situ* analysis of the thickness (*d*_A_), shear viscosity (*η*_A_) and shear elastic modulus (*µ*_A_) of the interface adlayer.

Areal mass density of the adlayer (*M*_A_^QCM^) can be calculated from the resulted uniform thickness *d*_A_ in case of a given effective mass density (*ρ*_A_):

5 $${M}_{A}^{QCM}={d}_{A}{\rho }_{A}$$

**Figure 6:**
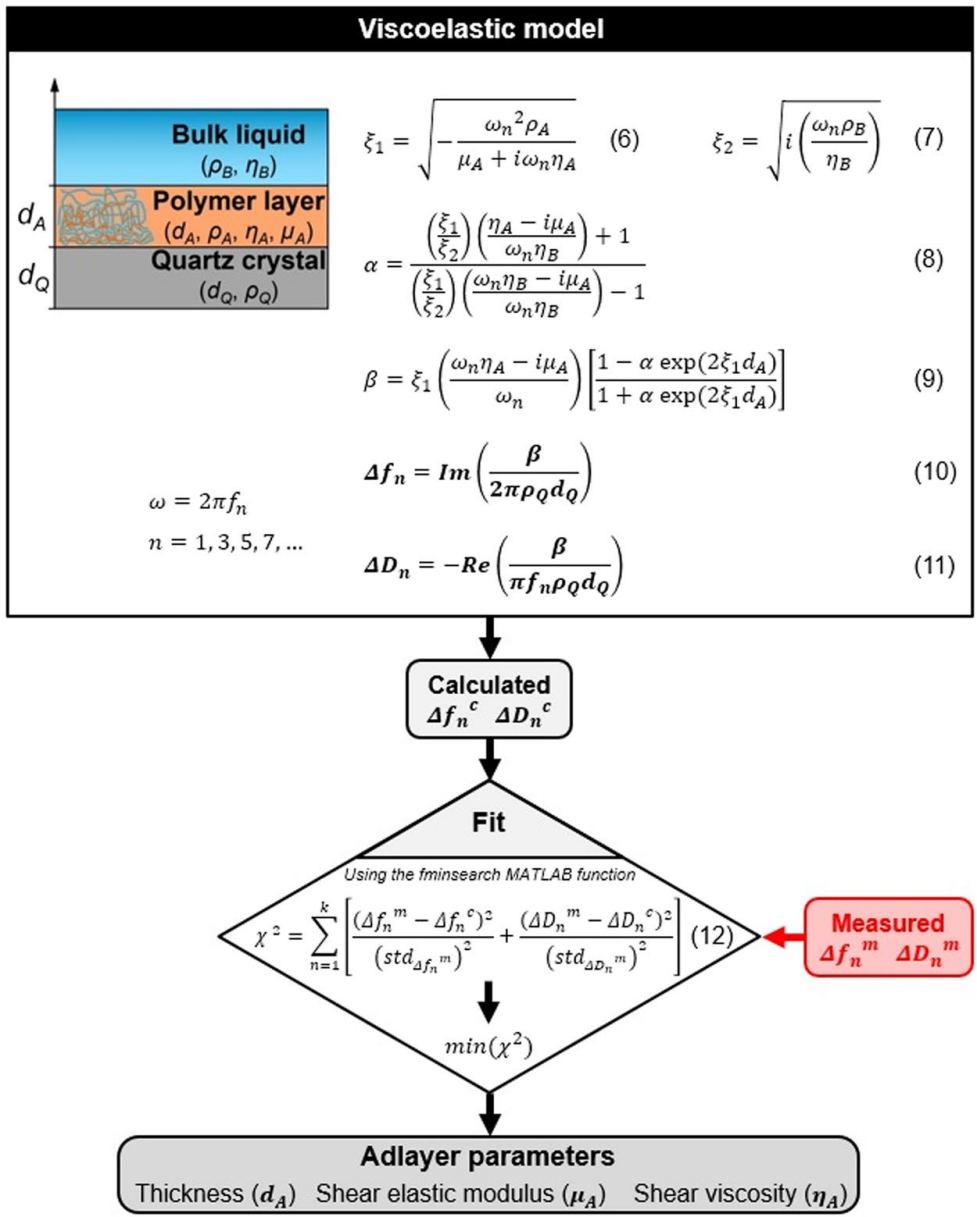
Schematic representation of the QCM data analysis code implemented in MATLAB. In the inset figure on the left upper side, one can see the structure of the applied model where the QCM chip was covered by one viscoelastic film consisted of the deposited polymer chains. The used equations of the Voigt-based model can be seen in the scheme^52^, where *ρ*_Q_ and *h*_Q_ are the density and thickness of the crystal respectively, *ρ*_B_ and *η*_B_ are the density and viscosity of the bulk liquid as well as *ω*_*n*_ is the angular frequency of the oscillation at a given *n* overtone. The hydrated thickness, density, shear viscosity and shear elastic modulus of the examined adlayer are represented by *d*_A_*, ρ*_A_*, η*_A_ and *µ*_A_, respectively. In the calculation of the error function *χ*^*2*^ the Δ*f*_*n*_^*m*^, Δ*f*_*n*_^*c*^ as well as Δ*D*_*n*_^*m*^, Δ*D*_*n*_^*c*^ are the measured and computed frequency and dissipations, respectively, the std values correspond to the standard deviations of the measured frequencies and dissipation at the baseline and *k* denotes the highest overtone number.

To calculate the viscoelastic parameters of the film, we developed an evaluation code and a graphical user interface (GUI) in MATLAB environment based on the viscoelastic model elaborated by Voinova and co-workers^52^, as it is also used in the commercial QTools program which is distributed for QCM-D instruments. An important aid was provided for the model implementation by a study of McNamara *et al*., which published a well-documented MATLAB code for predicting the viscoelastic parameters of films by the Voigt-based model with the purpose of giving support to experimental design in QCM measurements^57^. Our program takes the measured Δ*f* and Δ*D* values at the selected overtones for each measurement time and fits the parameters of the adlayer (*d*_A_, *η*_A_, *µ*_A_) using the MATLAB built-in *fminsearch* function to find the minimum of the sum of squares of the scaled errors^58^ (*χ*^2^) between the experimental and model Δ*f* and Δ*D* values by the simplex method. For the used model equations and schematic representation of the program see Fig. 6. The density and viscosity of the bulk liquid (buffer or pure water) were assumed to be 1000 kg/m^3^ and 1.0 mPa·s, respectively. The *ρ*_A_ density of the adlayer was also beforehand estimated and fixed at 1000 kg/m^3^.
